# The Effect of Seawater on Mortar Matrix Coated with Hybrid Nano-Silica-Modified Surface Protection Materials

**DOI:** 10.3390/polym14194080

**Published:** 2022-09-29

**Authors:** Yue Gu, Ruyan Fan, Kailun Xia, Kai Lyu, Zhenhua Wei, Mingzhi Guo

**Affiliations:** 1 College of Mechanics and Materials, Hohai University, Nanjing 210098, China; 2 College of Civil and Transportation Engineering, Hohai University, Nanjing 210098, China; 3 Department of Ocean Science and Engineering, Southern University of Science and Technology, Shenzhen 518055, China

**Keywords:** cement-based materials, nano-silica, surface treatment, seawater

## Abstract

Surface treatment technology is an effective method to reinforce the durability of concrete. In this study, cement-based materials containing industrial solid wastes were modified by hybrid nano-silica (HN), then applied as a novel surface protection material (SPM-HN). The effect of SPM-HN on surface hardness of mortar matrix exposed to seawater was investigated. Further, the microstructure was characterized by X-ray diffraction (XRD), Fourier transform infrared spectroscopy (FTIR), and mercury intrusion porosimetry (MIP). The results show SPM-HN could significantly enhance the surface hardness of matrix in seawater curing, and the rebound number is increased by 94%.The microstructure analysis demonstrates that the incorporation of HN inhibits the formation of ettringite, thaumasite, and Friedel’s salt. In addition, thermodynamic modeling shows the incorporation of hybrid nano-silica could generate more C-S-H, and decrease the maximum volume of Friedel’s salt when SPM is exposed to seawater. This research indicates SPM-HN can be applied as a concrete protective layer in the marine environment.

## 1. Introduction

Concrete is the most used construction material in civil engineering. However, the usage of concrete is facing a huge challenge, considering fact that it is responsible for 5~7% of global anthropogenic greenhouse gas emissions [1,2,3,4]. To make concrete more sustainable, a variety of solutions were proposed. One interesting alternative is to use surface treatment techniques to improve the durability of concrete, which might reduce carbon emissions caused by demolition and reconstruction [5,6,7,8].

Numerous researchers studied the use of nanoparticles in concrete surface treatment technologies, along with the advancement of nanotechnology. Sanchez et al. [9] explored a migration treatment of nano-silica for hardened mortar. It is found that nano-silica could infiltrate into the mortar by the electric field and perform the pozzolanic reaction. Hou et al. [10] reported that sparing the nano-silica could reduce the transport properties of the cement matrix, resulting from the alteration of pore structure. Javid et al. [11] investigated the influence of spraying nanoparticles (nano-silica, nano-halloysite, and nano-montmorillonite) on surface characteristics of concrete pavement, and found the effectiveness of nanoparticles in improving the abrasion and skid resistance of concrete surface. 

However, the form of applying nanoparticles mentioned above is the spraying method, which is the main form in the literature [12,13], while fewer studies focus on the indirect way. The drawbacks of the spraying method are the low penetration range and poor weatherability of the coating. The indirect way is modifying the surface protection materials by nanoparticles first, then coating it on the surface of the matrix. Compared with the former methodology, it has a better consistency with the matrix. More recently, Zhang et al. [14] conducted an attempt to use cement mortar with nano-silica as a type of surface protection material, which shows excellent integrity and interfacial bond strength. 

Common solid wastes such as fly ash and blast furnace slag could take the place of some cement when used as mineral admixtures in cementitious materials [15,16]. The utilization of these in surface protection materials could further magnify their value. In our previous work [17], nano-silica-modified cementitious materials were used as a type of coating against chloride ion attack. However, the waste utilization and seawater erosion have not been explored. In this study, a type of hybrid nano-silica-modified surface protection materials containing solid wastes was prepared, and its performance exposed when to seawater was investigated. The evaluated properties of surface protection materials including surface hardness, the mineral composition, and pore structure distribution of microstructure in specimens were examined using X-ray diffraction analysis, Fourier transform infrared spectroscopy analysis, and mercury intrusion porosimetry. The results highlight the advantages of hybrid nano-silica-modified cement mortar used as surface protection for materials exposed to seawater, which sheds new light for its application in the surface treatment area. 

## 2. Materials and Methods

### 2.1. Materials 

Ordinary Portland cement in accordance with Chinese standard GB175-2007 and industrial solid wastes (i.e., fly ash and blast furnace slag), were used as binders to prepare mixes. The chemical compositions of raw materials are listed in Table 1. Natural quartz sand with a fineness modulus within 2.3~3.0 was adopted as fine aggregate. Polycarboxylate superplasticizer produced by Jiangsu Sobute New Materials Co., Ltd. (Nanjing, China) was introduced as a dispersion agent to ensure the workability of cement composites.

**Figure 1:**
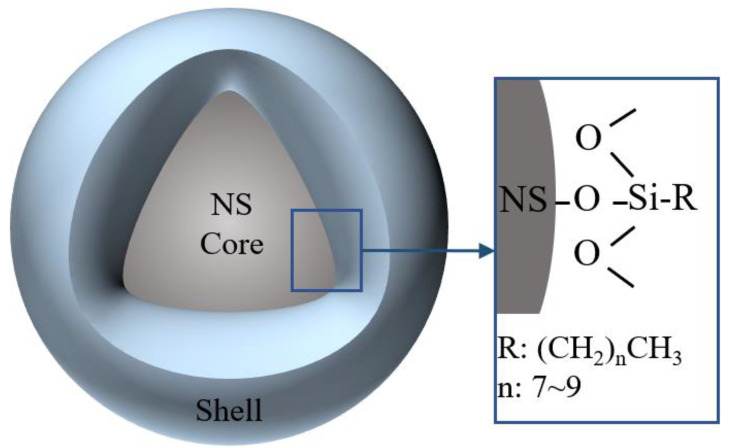
Schematic illustration of HN.

The commercially available organic–inorganic hybrid nanomaterials (HN) were provided by the Jiangsu Sobute New Materials Co., Ltd. According to the supplier, the inorganic constituent of HN is nano-silica, while organic component in HN is the aliphatic molecular group that accounts for 13% by weight. The diameter of the HN ranges between 30~60 nm. Figure 1 shows the schematic illustration of HN.

### 2.2. Specimen Preparation 

The hybrid nano-silica-modified mortar specimen was prepared as a surface protection material, and the matrix coated with it was marked as SPM-HN. The mass ratio of water-to-binder and binder-to-sand ratio were set as 2/5 and 1/3, respectively. For the binders, the mass ratio of cement: fly ash: slag was 0.70:0.15:0.15. The dosage of hybrid nano-silica was 1% by weight of the binders. For comparison, the specimen without hybrid nano-silica was also prepared, and matrix coated with it was marked as SPM-N. The mortar specimen with a water-to-cement ratio of 0.53 and binder-to-sand ratio of 0.33 was prepared as a matrix, marked as REF.

The matrix mortar was firstly fabricated, then cured for 1 d in a standard curing chamber (20 °C, 95%RH). After that, the surface of matrix was covered with SPM with a thickness of 20 mm. The specimens were demolded for 1 day after casting, then soaked in a simulated seawater environment until the test. The simulated seawater was prepared according to ASTM D114-2003, and its chemical composition is listed in Table 2.

### 2.3. Test Methods

#### 2.3.1. Rebound Surface Hardness 

The surface hardness of specimen was measured by the rebound test. The rebound numbers were recorded by using the rebound hammer hitting the surface of specimen according to GBT50315-2011. The nominal kinetic energy of the rebound hammer used is 0.196 J. For each surface, 10 points are randomly measured, and the average is taken after removing the maximum and minimum.

#### 2.3.2. X-ray Diffraction (XRD) 

The mineral phase assemblage of specimen after seawater corrosion was identified by X-ray diffraction analysis. XRD tests were conducted on powdered specimen mixtures after isopropyl alcohol solvent exchange at the desired age using a Bruker D8 Advance (Bruker Inc., Germany) diffractometer in a θ–θ configuration using Cu Kα radiation. The scanning range was 5~70 °C (2θ) with a scanning ratio of 5°/min.

#### 2.3.3. Fourier Transform Infrared Spectroscopy (FTIR) 

Fourier transform infrared spectroscopy (FTIR) analysis was performed on a FTIR workstation (Thermo Scientific, Waltham, MA, USA, Nicolet iS5). The powder sample was palletized with KBr, and the spectra were collected in the range of 400~4000 cm^−1^ at a resolution of 1 cm^−1^.

#### 2.3.4. Mercury Intrusion Porosimetry Test (MIP)

The pore structure of specimen after soaked in seawater for 28 d was detected by the Mercury intrusion porosimetry method. The MIP tests were performed on a Micromeritics AutoPore IV 9500 porosimeter (Micromeritics, Norcross, GA, USA), with a maximum pressure of up to 228 Mpa. Pore size ranging from 0.003 μm to 360 μm was recorded. The geometry of pore was assumed to be cylindrical. The sample with a thickness of about 3 mm was immersed in alcohol to stop cement hydration, and dried in an oven until constant weight before the test. 

#### 2.3.5. Thermodynamic Modeling 

Thermodynamic model calculations were carried out using the Gibbs free energy minimization software (GEMS) [18]. The thermodynamic properties of aqueous species, complexes, and solids were sourced from the default GMES-PSI database. Cemdata18 database [19] was further applied for interaction of cement-based materials and seawater.

## 3. Results and Discussion 

### 3.1. Surface Hardness Change

**Figure 2:**
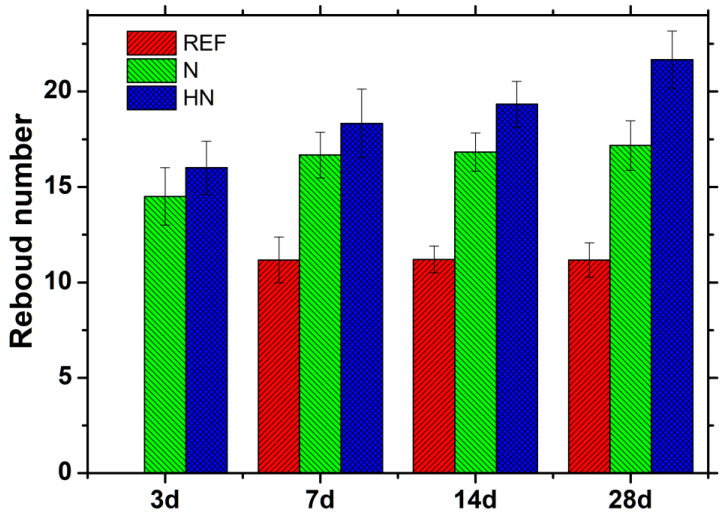
Surface rebound numbers of specimen exposed to seawater.

The surface rebound numbers are employed to give an indication of the surface hardness change in the specimen [20]. Figure 2 represents the surface rebound numbers of specimens exposed to seawater. As shown in Figure 2, in general, the surface hardness of SPM-HN is higher than SPM-N and REF at all ages. At 28 d, the rebound numbers of SPM-HN and SPM-N improve by 94.0% and 57.3%, respectively, compared with REF. This indicates that the introduction of hybrid nano-silica could generate an extra 40.3% increase. For REF, there is no value detected at 3 d, which means the hydration of binder ingredients is suppressed by seawater. From 3 d to 28 d, the rebound numbers are similar. This may imply that the cohesion between hydration production originates from the hydration of raw binder materials, to compensate for the destructive force caused by seawater erosion. For SPM-N, it reaches a high rebound number at day 3, while this value almost no longer increases after 7 days. For SPM-HN, its rebound maintains a slow growth over time. This observation indicates that the cohesion of the hydration product is stronger than the destruction by seawater. The hybrid nano-silica in SPM-HN could react with calcium hydroxide to form the C-S-H gels, as hydration continues [21]. The new C-S-H gels could block the pores produced by the crystallization of corrosion products, resulting in a denser microstructure. 

### 3.2. XRD Analysis 

**Figure 3:**
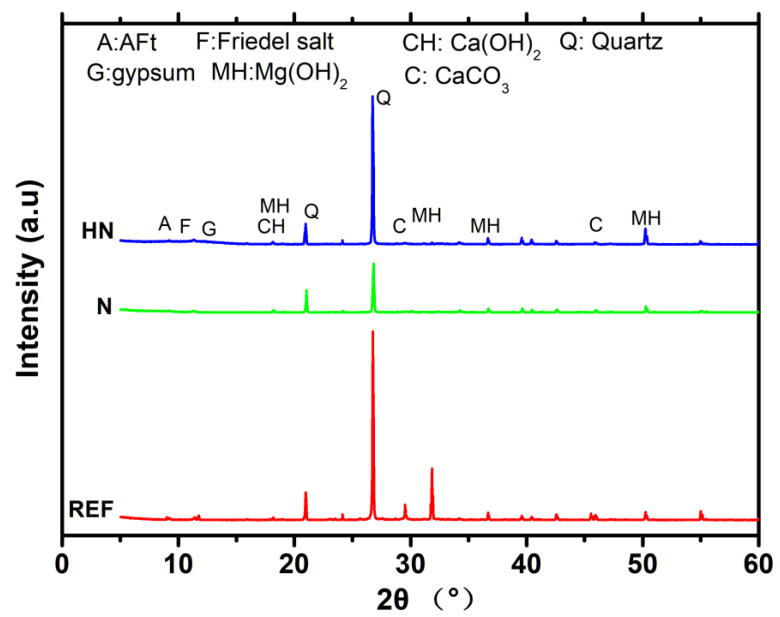
XRD patterns of specimen exposed to seawater for 28 d.

XRD analysis was conducted to acquire phase assemblages of specimens. Figure 3 shows the XRD patterns of specimens exposed to seawater for 28 d. It can be seen from Figure 2 that, for all three specimens, the main detected crystalline products are similar, including ettringite (AFt), Friedel’s salt, Ca(OH)_2_, Mg(OH)_2_, CaCO_3,_ and quartz. These products are consistent with experimental observation reported by De Weerdt [22]. Although the powder specimens are sieved to remove quartz, the peak of quartz is still high, which may be related to the residues during the grinding process. The peak around 32° is attributed to Mg(OH)_2_, and the intensity of SPM-HN here is significantly lower than that of the matrix. The peak around 29° is related to CaCO_3_, and SPM-N also has a lower intensity than REF. Mg(OH)_2_ and CaCO_3_ are the principal crystallized products, due to the entry and reaction of erosion ions in seawater. These findings indicate that SPM-HN could inhibit the formation of calcium carbonate and magnesium hydroxide, which may be attributed to its dense microstructure with low porosity. 

**Figure 4:**
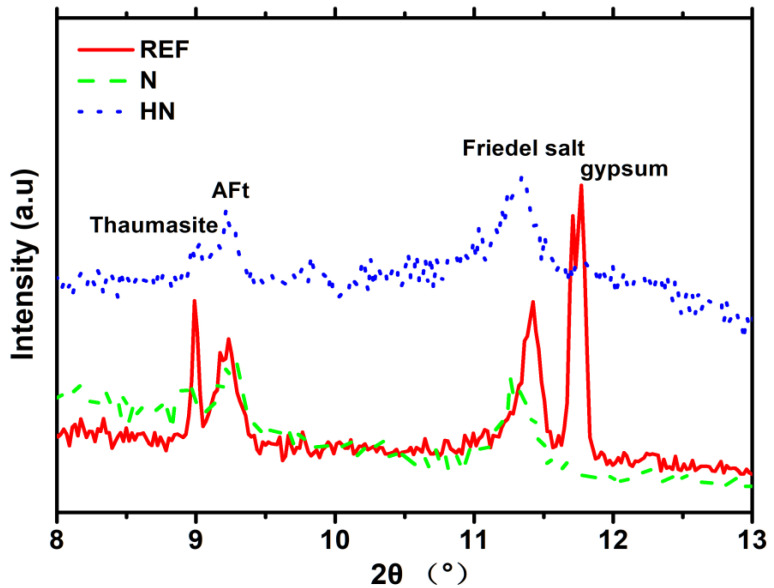
XRD patterns of specimen near the peak of Friedel’s salt.

Owing to the fact that ettringite and Friedel’s salt are characteristic products of the erosion of chloride ion and sulfate ion in seawater, the peak range containing these substances in Figure 3 is selected and highlighted, as shown in Figure 4. From Figure 4, the peaks around 9° and 11.5° are ascribed to AFt and Friedel’s salt, respectively. For the peak of AFt, all three specimens have a similar intensity. There may be two reasons: (i) sulfate attack is not severe; (ii) sulfate may be easier to appear in other crystalline products, such as gypsum. The presence of gypsum in REF seems to support the second inference. It is noted that there is no gypsum present in SPM-HN and SPM-N. For the peak of Friedel’s salt, the intensity of SPM-HN is lower than REF, while higher than SPM-N. Usually, chloride ion would bond in the hardened cementitious composites in forms of chemical bound and physical adsorption. The formation of Friedel’s salt is the reflection of chemical bonding. By comparison of Friedel’s salt peaks, it is shown that SPM-HN has a weaker chemical chloride binding capacity than REF, while it is slightly stronger than SPM-N. Another interesting observation is about thaumasite. It can be seen from Figure 4 that there is a strong peak before the peak of AFt in the curve of REF. It provides the evidence for the existence of thaumasite. According to the report by De Weerdt [23], thaumasite would emergence when the ratio of seawater-to-cement is under about 1 L/100 g. Unlike REF, the peak of thaumasite does not appear in the curve of the SPM-HN, and this peak is also very slight in SPM-N. This indicates SPM-HN could hinder the crystallization of thaumasite. In summary, compared to REF, both SPM-HN and SPM-N could suppress the formation of gypsum and thaumasite when exposed to seawater. The chloride-binding capacity of SPM-HN is stronger than SPM-N, while weaker compared to REF. 

### 3.3. FTIR Analysis 

**Figure 5:**
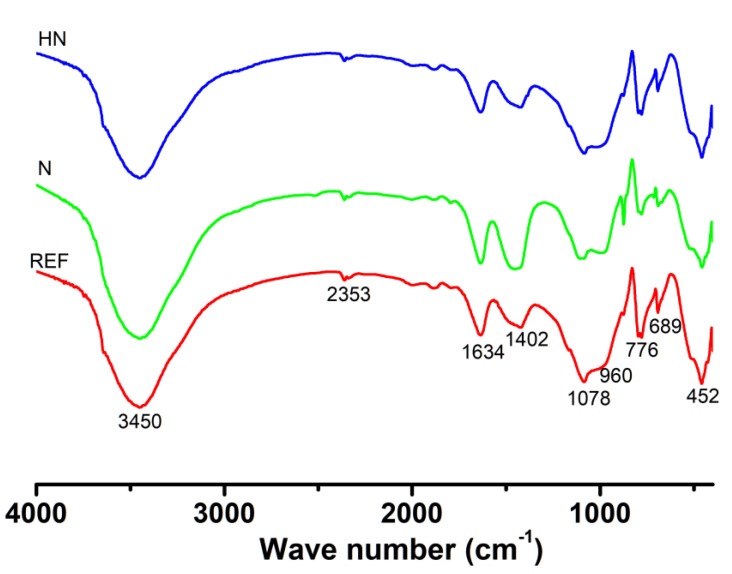
FTIR patterns of specimen exposed to seawater for 28 d.

Figure 5 displays the FTIR patterns of specimen exposed to seawater for 28 d. The broad band around 3450 cm^−1^ is associated with the stretching vibration of H-O-H, which is mainly caused by portlandite in the products. A small band at 1634 cm^−1^ is associated with the bending vibration of H-O-H, corresponding to the chemical bonding water. The peak around 1402 cm^−1^ is related to the symmetric stretching vibration of C-O, verifying the existence of calcium carbonate. The SPM-HN has a relatively low peak, which suggests a lower amount of calcium carbonate compared with SPM-N. The band located in 776 cm^−1^ could be attributed to in-plane bending vibration of $SO_{4}^{2-}$, which provides the evidence for the presence of gypsum or Aft. The band at 452 cm^−1^ is characteristic of the bending vibration triggered by Si–O in silicate minerals. The peak intensities of the three specimens are similar, indicating that there is no significant increase in silicate minerals in SPM-HN. The range between 950 cm^−1^ and 1200 cm^−1^, referred to as fingerprint zone, could offer some information on polymerization of silicate group in C-S-H. The band at 960 cm^−1^ is associated with sites in the Q_2_ tetrahedron and would shift when the Q_3_ tetrahedron dominates C-S-H. For all three specimens, the peak intensity at high frequency is greater than that at low frequency in the fingerprint zone, indicating a decrease in Ca/Si of C-S-H. This phenomenon may be attributed to the decalcification process of specimens when they are exposed to the seawater. Compared with REF, the SPM-HN has a lower intensity at high frequency. It indicates that there are more C-S-Hs with a high Ca/Si ratio in SPM-HN. 

### 3.4. MIP Analysis 

**Figure 6:**
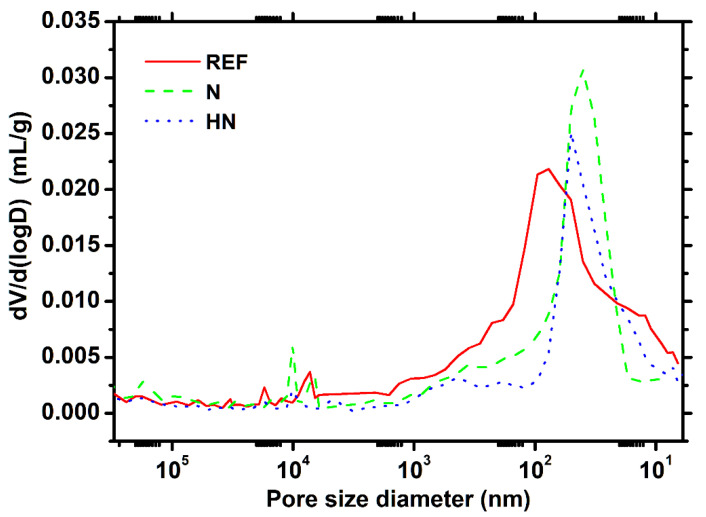
Differential pore volume profiles of specimens exposed to seawater for 28 d.

The pore structure of cementitious materials is a vital characteristic that exerts a significant impact on surface hardness. To quantitatively evaluate the pore structure of specimens, the MIP test was performed. Figure 6 shows the differential pore volume profiles a of specimens exposed to seawater for 28 d. 

It can be seen from Figure 6 that there are several peaks that appear in the curves. These peaks are the reflection of different types of pores. According to the method proposed by refs. [24,25], the pores in cementitious materials are classified as three types in terms of their sizes: (1) gel pore (<0.01 μm); (2) medium capillary pore (0.01~0.05 μm); and (3) large capillary pore (0.05 μm~10 μm). 

For all specimens, the strongest peak is found in the medium capillary pore region. The position of SPM-HN is comparable to SPM-N, while lower than REF. This implies medium capillary pores in SPM-HN and SPM-N have lower connectivity compared with REF. It should be noticed that whereas SPM-HN does not have any small peaks in the region of the big capillary pore, REF and SPM-N do. It implies that SPM-HN has a minimal volume of large capillary pores.

Compared with REF, the total porosity of SPM-N and that of SPM-HN decrease by 8.2% and 22.9%, respectively. The total porosity should be mainly determined by two factors. On one hand, the porosity slowly declines with the hydration of binder materials. On the other hand, some corrosion products are formed inside the specimens due to the attack of ions in seawater. This process causes great expansion pressure inside the specimens that generate micro-cracks, resulting in the growth of the porosity. The SPM-HN has the lowest total porosity, which further demonstrates its excellent ability to resist seawater erosion.

**Figure 7:**
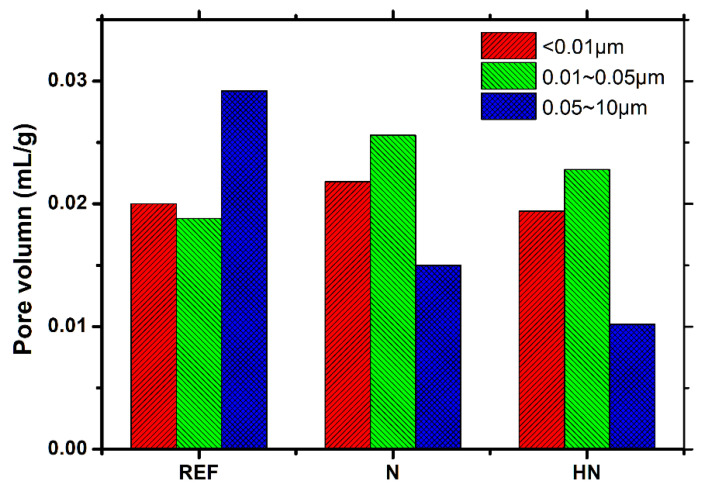
Pore size distribution of specimens exposed to seawater for 28 d.

The data in Figure 6 are extracted, then calculations are made based on the previously specified categorization interval in order to quantitatively compare the relative concentration of three different types of pores. The outcome is represented graphically in Figure 7. According to Figure 7, the medium capillary pore, followed by the gel pore, has the highest relative volume content among the three types of pores for specimens with SPM. It is observed that specimens with SPM show a considerable reduction in large capillary pores. In particular, the SPM-N and SPM-HN decrease by 48.6% and 65.1%, respectively. It is clear that the pore-refining effect in SPM-HN is more pronounced. The large capillary pore is closely related to the permeability and penetration of exogenous ions into cementitious materials [26,27]. A lower volume of this type of pore in SPM-HN contributes to reducing the risk of erosion by seawater. 

### 3.5. Thermodynamic Modeling

Thermodynamic modeling was used to predict phase assemblages of NS-incorporated blends exposed to seawater. The dose of hybrid nano-silica is 1 wt.% by weight of the binders, as stated in Section 2.2. However, considering the agglomeration trends of NS, it is difficult for NS to acquire an ideal uniform dispersion state in hardened cement paste, which implies that the enrichments of NS may arise in blends, resulting in a greater local concentration. Three doses are determined based on the above hypothetical scenarios: 0 wt.%, 1 wt.%, and 5 wt.%.

**Figure 8:**
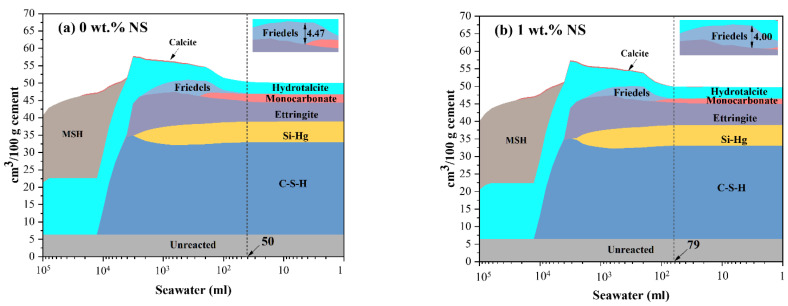

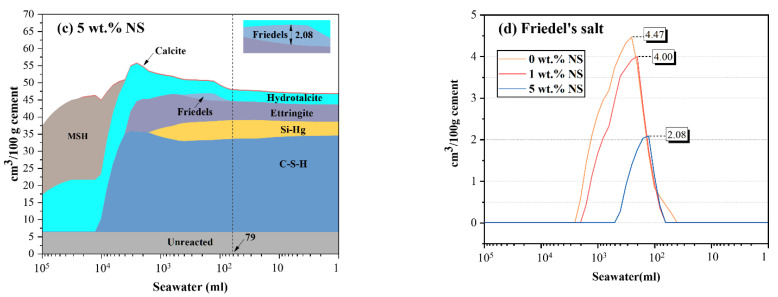
Phase assemblages in the blends incorporated with different content of NS upon exposure to increasing amount of seawater; (**a**–**c**) total phase assemblages; (**d**) volumes of Friedel’s salt.

Figure 8a shows the evolution of phase volumes as seawater progresses in blends. Blends contain C-S-H, siliceous hydrogarnet (Si–Hg), ettringite, monocarbonate, and hydrotalcite before being exposed to seawater. The consumption of monocarbonate is noticed initially after contact with roughly 50 mL saltwater, followed by the production of Friedel’s salt. It is worth mentioning that after reaching a maximum capacity of 4.47 cm^3^, the Friedel’s salt progressively disintegrates. The development of C-S-H, ettringite, and hydrotalcite increases the overall volume of solid phase throughout this process. After that, when the concentration of saltwater rises, ettringite and C-S-H dissolve, while MSH and calcite develop, resulting in a reduction in the total volume of solid phase.

Phase compositions of blends with NS exposure to seawater are shown in Figure 8b,c. It is noted that the initial volumes of monocarbonate and Si–Hg decrease remarkably, while that of C-S-H and calcite increase. (The specific values can be seen in Table 3). These chemical changes imply that calcite and C-S-H are more stable than monocarbonate and Si–Hg, if there is adequate silicon supply. It is possible that silicon obtained from NS might occupy calcium derived from monocarbonate (3CaO·Al_2_O_3_·CaCO_3_·11H_3_O), causing C-S-H to develop and the release of Al_2_O_3_ and CaCO_3_. This might explain the rise in ettringite volume in blends containing 1 wt% NS and the production of calcite in blends containing 5 wt% NS.

A similar reaction between SiO_2_ and Si–Hg (3CaO·xAl_2_O_3_·(1 − x)Fe_2_O_3_·0.84SiO_2_·4.32H_2_O) can be inferred. In such a situation, NS could serve as silicon sources and calcium capturer. Therefore, in addition to portlandite, NS also have potential in reacting with other metastable calcareous hydrates to produce C-S-H.

Similar patterns in phase composition change can be seen in Figure 8b,c. Furthermore, when including 1 wt.% or 5 wt.% NS, the threshold of seawater concentration that causes the production of Friedel’s salt shifts to 79 mL. This finding suggests that NS may be able to delay the formation of Friedel’s salt. NS can also impact the content of created Friedel’s salt, as illustrated in Figure 8d. The maximum volume of Friedel’s salt in blends decreases to 4.00 cm^3^ and 2.08 cm^3^, when 1 wt.% and 5 wt.% NS are added, respectively. The decrease might be attributed to the calcium uptake by NS, given the chemical components of Friedel’s salt (3CaO·Al_2_O_3_·CaCl_2_·10H_2_O). This suggests that in the absence of calcium, the presence of NS may obstruct the formation of Friedel’s salt.

## 4. Conclusions

In this work, a type of hybrid nano-silica-modified cement mortar containing industrial solid wastes (SPM-HN) was prepared and used as a surface protection material (SPM). The influence of SPM-HN on surface hardness of the mortar matrix (REF) exposed to seawater was studied compared with normal surface protection materials without hybrid nano-silica (SPM-N). Based on the experimental results, the following principal conclusions can be summarized:

1. The addition of hybrid nano-silica beneficially contributes to the improvement in surface hardness of surface protection materials exposed to seawater. After exposed to seawater for 28 d, the rebound number of SPM-HN increases by 94.0%, while and that of SPM-N increases by 57.3%, compared with REF;
2. The incorporation of HN helps to inhibit the formation of ettringite, thaumasite, and maintains more C-S-H with a high Ca/Si ratio in the microstructure of SPM. However, it weakens the ability of chemically binding chloride of SPM;
3. A lower total porosity and large capillary pore volume in SPM-HN are observed. This highlights the pore-refinement effect of hybrid nanomaterials;
4. The results of thermodynamic modeling show that the incorporation of nano-silica could generate more C-S-H, delay the formation of Friedel’s salt, and decrease the maximum volume of Friedel’s salt when SPM are exposed to seawater.

Further study on the effect of hybrid nano-silica-modified surface protection materials on other performance of cementitious matrix is still ongoing. This study highlights the potential application value of SPM-HN in marine environments.

## Figures and Tables

**Table 1:** Chemical composition of raw materials.

	CaO	SiO_2_	Al_2_O_3_	MgO	Fe_2_O_3_	SO_3_	K_2_O	Na_2_O
Cement	62.83	20.5	5.61	1.70	3.84	3.07	1.31	0.21
Fly ash	3.39	57.53	28.34	1.22	4.07	0.74	2.51	1.06
Slag	26.51	46.29	7.48	10.46	5.05	0.25	1.76	0.09

**Table 2:** Chemical composition of simulated seawater (kg/m^3^).

NaCl	NaSO_4_	MgCl_2_·6H_2_O	CaCl_2_	KCl_2_
24.5	4.1	11.1	1.2	0.7

**Table 3:** Volumes of hydrates before exposure to seawater.

Blends	Volumes of Hydrates (cm^3^/100 g Cement)					
	Calcite	Hydrotalcite	Monocarbonate	Ettringite	Si–Hg	C-S-H
0 wt.% NS	0.00	3.24	2.40	5.44	5.94	26.58
1 wt.% NS	0.00	3.20	1.62	5.97	5.85	26.66
5 wt.% NS	0.32	3.04	0.00	5.02	4.04	28.08

## Data Availability

The data used to support the findings of this study are available from the corresponding author upon request.
